# Supplementary material for: Magnetotactic bacteria in a droplet self-assemble into a rotary motor
Source: Nat Commun. 2019 Nov 8;10:5082. doi: 10.1038/s41467-019-13031-6 (PMC6841940; doi:10.1038/s41467-019-13031-6)
Supplement: Supplementary file 1 — Supplementary Information [file 41467_2019_13031_MOESM1_ESM.pdf]

Supplementary Information: Magnetotactic bacteria in a droplet self-assemble into a rotary motor

Benoit Vincenti,[1] Gabriel Ramos,[2] Maria Luisa Cordero,[2] Carine Douarche,[3] Rodrigo Soto,[2] and Eric Clement[1]

[1]*Laboratoire PMMH, UMR 7636 CNRS-ESPCI-Sorbonne Université-Université Paris Diderot, 7-9 quai Saint-Bernard, 75005 Paris, France.*

[2]*Departamento de Física, FCFM, Universidad de Chile, Av. Blanco Encalada 2008, Santiago, Chile.*

[3]*Laboratoire FAST, Univ. Paris-Sud, CNRS, Universitté Paris-Saclay, F-91405, Orsay, France.*

Supplementary Note 1. Hydrodynamic model of a sphere rotating in a fluid close to a surface

To estimate the torque exerted by a droplet of radius R on the oil, we use an hydrodynamic model of a sphere of radius R rotating in a fluid of viscosity $\eta = 3 \times 10^{-3}$ Pa.s (value for hexadecane oil at 25°C). The droplets being sat on the bottom plate of the pool, we have to take into account, in our model, the hydrodynamic image of the rotating sphere with respect to the bottom plate (see Supplementary Fig. 1 (a-b)). The flow created by a sphere of radius R, rotating thanks to a torque τ, reads, in the bulk :

$$V_\theta^{\text{oil},1}(r,\phi) = \frac{\tau \sin\phi}{8\pi\eta r^2}, \tag{1}$$

where ϕ is the azimuthal angle, $\phi = \pi/2$ at the equator of the droplet.

The correction due to the bottom wall corresponds to the superimposition of the flow in Eq. (1) and the flow created by the mirror image of the sphere with respect to the wall, which counter-rotates with respect to the real droplet rotation. The flow created by the mirror droplet at the equatorial plane of the real droplet is :

$$V_\theta^{\text{oil},2}(r',\phi') = -\frac{\tau \sin\phi'}{8\pi\eta (r')^2}, \tag{2}$$

with $r' = \sqrt{r^2 + 4R^2}$ and $\phi' = \pi - \arcsin\left(\frac{r}{r'}\right)$. Then, $\sin\phi' = \frac{r}{\sqrt{r^2+4R^2}}$ and the total flow at the equatorial plane of the droplet reads :

$$V_\theta^{\text{oil}}(r) = V_\theta^{\text{oil},1}(r,\phi) + V_\theta^{\text{oil},2}(r',\phi'),$$

$$\Rightarrow V_\theta^{\text{oil}}(r) = \frac{\tau}{8\pi\eta R^2}\left(\left(\frac{R}{r}\right)^2 - \underbrace{\frac{r/R}{((r/R)^2+4)^{3/2}}}_{\text{corrective term due to the bottom wall}}\right), \tag{3}$$

where the corrective term due to the bottom wall is emphasized.

The experimental flow field is very well captured by this hydrodynamic model, as shown on Supplementary Fig. 1 (b).

Supplementary Note 2. Alignment of magnetotactic bacteria with surfaces under constant magnetic field

We observed single bacteria inside droplets for dilute bacterial suspensions. We observed that bacteria align partially with the droplet boundary in the presence of a magnetic field, wobbling between the magnetic field direction and the local droplet interface. An image sequence (see Supplementary Movie 8) illustrates this particular motion and Supplementary Fig. 2 shows the average motion of one bacterium along the droplet boundary.

Supplementary Note 3. MTB velocity distribution and emulsion preparation

The MTB are grown and prepared as indicated in the Methods part of the article. Here are some details of the bacteria motion characteristics and of the emulsion preparation.

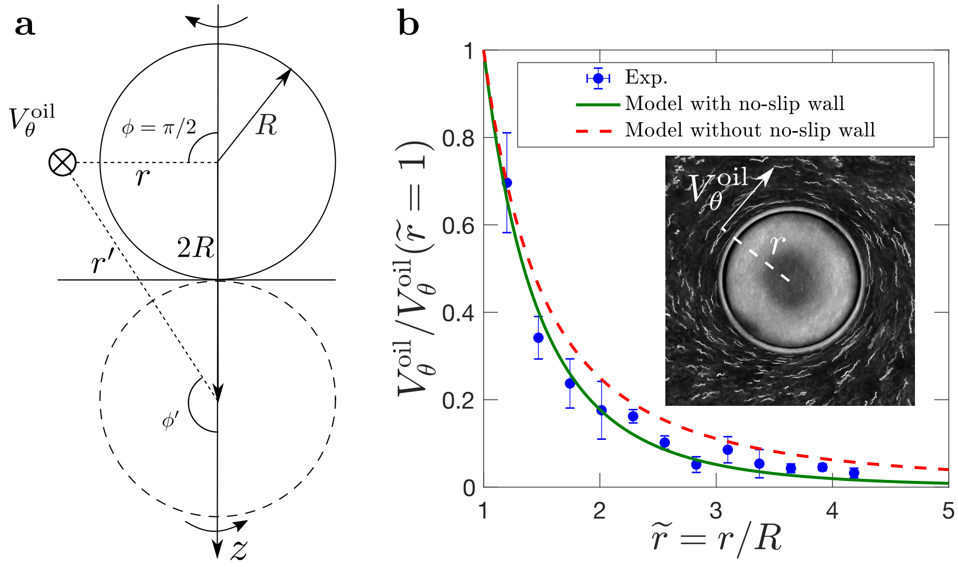

Supplementary Figure 1. Hydrodynamic model of a rotating sphere - application to the estimation of the torque exerted by one droplet on the oil. (a) Definition of the model and hydrodynamic correction due to the presence of the bottom plate of the pool. (b) Test of the hydrodynamic model on experimental data. Dotted line: model without corrective term accounting for the presence of the bottom plate. Solid line: model with corrective term.

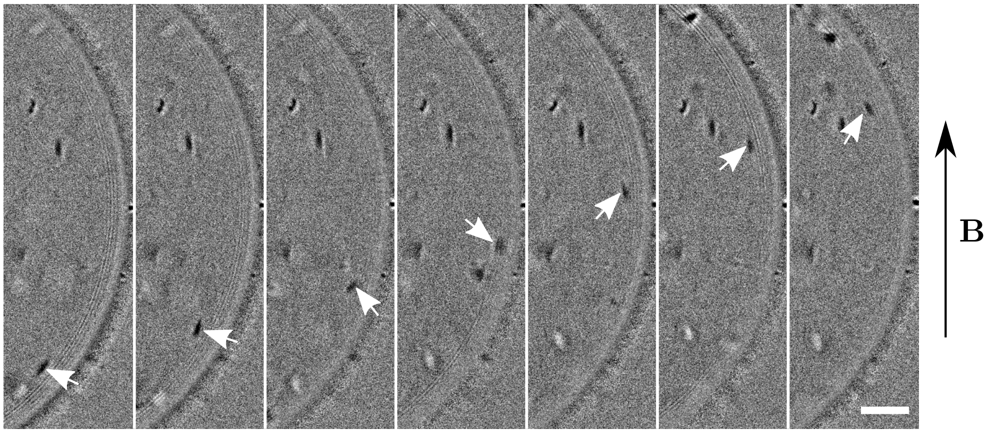

Supplementary Figure 2. Image sequence of bacteria motion along a droplet interface. A bacterium moves along a droplet interface while a magnetic field of 2 mT is applied. The white scale bar is 10 µm and images are separated by 0.2 s. In average, the bacterium moves with its body aligned with the droplet boundary. The Supplementary Movie 8 shows details about this temporal sequence: the bacterium exhibits a wobbling motion, aligning its body along the magnetic field direction and aligning with the local droplet tangent sequentially.

Before producing the emulsion, bacteria are always harvested after their growth to an optical density OD= 0.12±0.02. Their motility are always checked quantitatively by performing tracking of single bacteria at ×40 magnification. The velocity distribution (see Supplementary Fig. 3) is bimodal with two peaks located at 20 and 40 µm s^{-1}, reminiscent from their reversal motion sequence. This distribution is quantitatively reproducible using our growth protocol.

Concerning the emulsion preparation, we fill one 1.5 mL-eppendorf with 500 µL of hexadecane oil. A small amount of bacteria suspension (typically 10 µL) is added to the oil. Then, the eppendorf is gently shaked and a part of the emulsion (65 µL) is rapidly extracted and placed inside a pool composed by a double sided tape and a cover-glass. The pool is then sealed with a top cover-glass in such a way to avoid bubble formations.

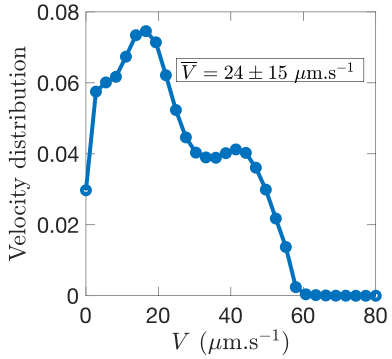

Supplementary Figure 3. Velocity distribution of the MTB after growth (these data are an average for a series of 6 growing sequences corresponding to ~6,000 tracked bacteria) .

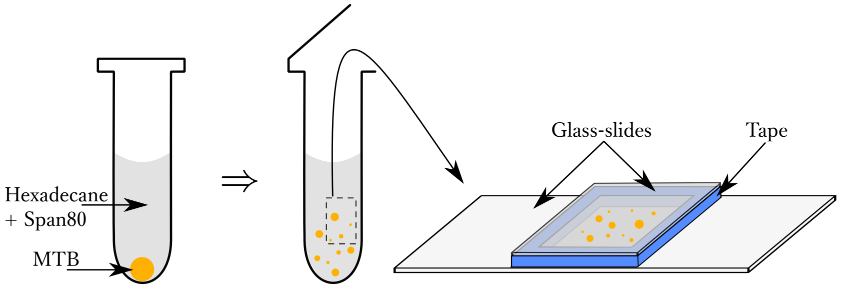

Supplementary Figure 4. Emulsion preparation protocol. We first stir hexadecane oil and MTB suspension. Then, we harvest a sample of the emulsion and transfer it into the experimental pool composed of two glass-slides and a GeneFrame tape.

Supplementary Note 4. Proof that the total circulation produced by a torque-free swimmer vanishes: detailed version

A torque and force free swimmer, produces on the far field (distances larger than the body length) a flow that is well described by that of a force dipole. For a swimmer located at $\mathbf{r}_0$, with director $\hat{\mathbf{n}}$ and force dipole intensity p, the produced velocity field is

$$u_i(\mathbf{r}) = -\frac{p}{8\pi\eta}\frac{x_i}{|\mathbf{x}|^3}\left(\delta_{jk} - \frac{x_j x_k}{|\mathbf{x}|^2}\right)n_j n_k, \tag{4}$$

where η is the fluid viscosity, $\mathbf{x} = \mathbf{r} - \mathbf{r}_0$, δ_{ik} is the Kronecker delta, and Einstein notation has been used for repeated indices. Note that the velocity field is radial ($\mathbf{u}/\!\!/\mathbf{x}$) with and intensity that depends on the angle. The circulation on a path γ, which is entirely contained in a plane oriented in the $\hat{\mathbf{z}}$ direction (see Supplementary Fig. 6), is

$$\Gamma_{z,\gamma} = \oint_\gamma \mathbf{u}\cdot d\mathbf{r} = \int_{A_\gamma} dA\,\omega_z, \tag{5}$$

where we have used Stokes theorem, A_γ is the area enclosed by the path, and ω is the vorticity field

$$\omega = \nabla\times\mathbf{u} = \frac{3p}{4\pi\eta}\frac{(\hat{\mathbf{n}}\times\mathbf{x})(\hat{\mathbf{n}}\cdot\mathbf{x})}{|\mathbf{x}|^5}. \tag{6}$$

In general, $\Gamma_{z,\gamma}$ will be different from zero (see Supplementary Fig. 5), but here we will show that if the paths are organized in parallel circles around a sphere of radius R, the total circulation vanishes.

First, the total circulation can be written as the integral over the sphere volume of the vorticity. Indeed,

$$\Gamma_z = \int dz\,\Gamma_{z,\gamma} = \int dz \int_{A_\gamma} dA\,\omega_z = \int_V dV\,\omega_z. \tag{7}$$

It is, then, direct to extend the definition for paths oriented on any direction

$$\boldsymbol{\Gamma} = \int_V dV\,\omega. \tag{8}$$

Second, we transform the volume integral into one over the surface of the sphere. For this, we note that

$$\omega_i = \frac{3p}{4\pi\eta}\epsilon_{ijk}n_j n_m\frac{x_k x_m}{|\mathbf{x}|^5}, \tag{9}$$

$$= \frac{p}{4\pi\eta}\epsilon_{ijk}n_j n_m\left(\frac{\partial^2}{\partial x_k\partial x_m}\frac{1}{|\mathbf{x}|} + \frac{\delta_{km}}{|\mathbf{x}|^3}\right), \tag{10}$$

$$= \frac{p}{4\pi\eta}\epsilon_{ijk}n_j n_m\frac{\partial^2}{\partial x_k\partial x_m}\frac{1}{|\mathbf{x}|}, \tag{11}$$

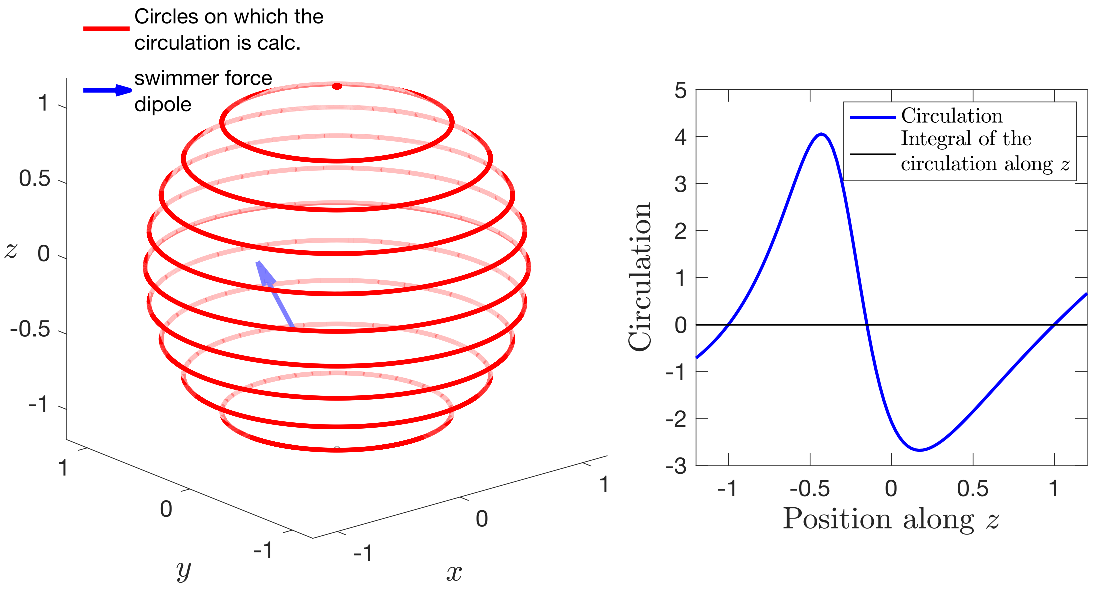

Supplementary Figure 5. Left panel: Representations of a force dipole located in a fixed point inside a sphere and some of the circles used in the circulation calculations. The circles are chosen parallel to the (x, y) plane without loss of generality and are of dimensions such that they altogether enclose a sphere of radius 1.2. The position and the orientation of the swimmer were chosen randomly inside a sphere of radius 1. Right panel: Circulation computed on each of the circles as a function of the vertical z position. The integral of the circulation along z is zero even if the circulation can be locally non-zero. This indicates that the sphere of fluid enclosing the micro-swimmer does not rotate globally, because no net torque is applied on it.

where ϵ_{ijk} is the Levi-Civita tensor and from going form the second to the third line we use that the cross product of equal vectors vanishes. It is possible to use the divergence theorem to obtain

$$\Gamma_i = \frac{p}{4\pi\eta}\epsilon_{ijk}n_j n_m \int_S dS_m \frac{\partial}{\partial x_k}\frac{1}{|\mathbf{x}|}, \tag{12}$$

$$= -\frac{p}{4\pi\eta}\epsilon_{ijk}n_j n_m \int_S dS_m \frac{x_k}{|\mathbf{x}|^3}. \tag{13}$$

The integral $T_{ij} = \int_S dS_j\, x_i |\mathbf{x}|^{-3}$ can be evaluated in spherical coordinates. Choosing integration axis such that $\mathbf{r}_0 = z_0\hat{\mathbf{z}}$, it is direct to verify that T_{ij} is diagonal. Furthermore, the diagonal components are

$$T_{xx} = T_{yy} = R^2 \int_0^\pi d\theta \frac{\pi \sin^3\theta}{(1 + z_0^2 - 2z_0\cos\theta)^{3/2}} = \frac{4\pi R^2}{3}, \tag{14}$$

$$T_{zz} = R^2 \int_0^\pi d\theta \frac{2\pi\cos\theta\sin\theta(\cos\theta - z_0)}{(1 + z_0^2 - 2z_0\cos\theta)^{3/2}} = \frac{4\pi R^2}{3}, \tag{15}$$

implying that it is an isotropic tensor, which can be expressed in any axes as $T_{ij} = 4\pi R^2\delta_{ij}/3$. Substituting this result in (13) implies that $\Gamma_i = 0$, concluding the proof.

For a collection of swimmers, the total induced flow is the sum of those produced by each of them. This linearity, valid for low Reynolds flows, implies that even though the circulation in a particular plane can be finite, its integral on z must vanish. As a consequence, for any distribution of torque-free swimmers in the sphere, the circulation must change sign when measured on different z planes.

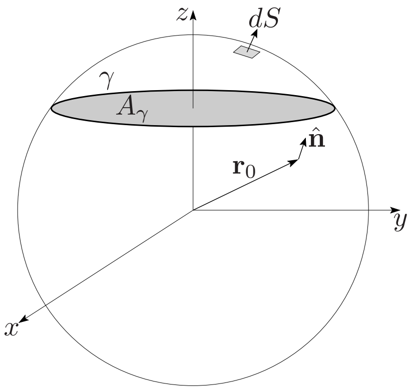

Supplementary Figure 6. Scheme used for the proof. The swimmer is located on any position $\mathbf{r}_0$ in the interior of the sphere of radius R. The path γ is a circle, tangent to the sphere, encircling the area A_γ, which is oriented along the z axis. The total circulation (7) is obtained integrating the circulation $\Gamma_{z,\gamma}$ for all vertical positions of the path, from $-R$ to R. The integral in (13) is done over the surface of the sphere with area elements dS.
