# Supplementary material for: Magnetotactic bacteria in a droplet self-assemble into a rotary motor
Source: Nat Commun. 2019 Nov 8;10:5082. doi: 10.1038/s41467-019-13031-6 (PMC6841940; doi:10.1038/s41467-019-13031-6)
Supplement: Supplementary file 3 — Description of Additional Supplementary Files [file 41467_2019_13031_MOESM3_ESM.pdf]

Description of Additional Supplementary Files

File Name: Supplementary Movie 1
Description: **Emulsion observation.** Phase-contrast images (x10 magnification) of a crowded emulsion prepared at a cell density n~1017 bact m−3 . The white scale bar is 100 μm. Under the presence of a magnetic field of 4 mT, in the direction indicated by the arrow, the suspension inside the droplets clearly rotates with a vorticity oriented perpendicularly to the image plane. Almost all the droplets rotate CW except one droplet, located at the bottom right-hand side of the movie, which rotates CCW.

File Name: Supplementary Movie 2
Description: **Bacteria gather at the poles.** Phase-contrast images (x40 magnification, average intensity is subtracted from the movie) of a suspension of magnetotactic bacteria at low cell density, n~1014 bact m−3 at the equatorial plane of a droplet of radius R = 43 μm, under the presence of a magnetic field of 4 mT (direction indicated by the arrow). White scale bar is 10 μm. Bacteria gather at the poles of the droplet.

File Name: Supplementary Movie 3
Description: **Bacteria form hydrodynamic jets at the poles.** Phase-contrast images (x40 magnification, average intensity is subtracted from the movie) of a suspension of magnetotactic bacteria at intermediate cell density, n~1015 bact m−3 at the equatorial plane of a droplet of radius R = 89 μm, under the presence of a magnetic field of 4 mT (direction indicated by the arrow). White scale bar is 20 μm. Bacteria gather at the poles of the droplet and form hydrodynamic jets expelling bacteria from the pole regions and creating recirculation flows.

File Name: Supplementary Movie 4
Description: **Bacteria self-assemble into a rotary motor.** Phase-contrast images (x40 magnification, average intensity is subtracted from the movie) of a suspension of magnetotactic bacteria at high cell density, n~1017 bact m−3 at the equatorial plane of a droplet of radius R = 55 μm, under the presence of a magnetic field of 4 mT (direction indicated by the arrow). White scale bar is 20 μm. When no magnetic field is set (beginning of the movie), bacteria move in a random way with no large scale collective motion. Once a magnetic field is set, bacteria self-assemble and form a vortex at the center of the droplet. Some bacteria countercirculate close to the poles of the droplet. Passive tracers are present in the outer fluid and are advected in average in the same direction as the core rotation. Tracers counter-circulate close to poles, mirroring the inner counter flows.

File Name: Supplementary Movie 5
Description: **Bacteria rotate above the equatorial plane**. Phase-contrast images (x40 magnification, average intensity is subtracted from the movie) of a suspension of magnetotactic bacteria at a plane parallel and above the equatorial plane of a droplet for a high cell density n~1017 bact m−3 and under the presence of a magnetic field (arrow). White scale bar is 20 μm. The droplet is identical to the one in Supplementary Movie 4. Bacteria rotate in the same direction (CW) than in the equatorial plane.

File Name: Supplementary Movie 6
Description: **Bacteria rotate below the equatorial plane**. Phase-contrast images (x40 magnification, average intensity is subtracted from the movie) of a suspension of magnetotactic bacteria at a plane parallel and below the equatorial plane of a droplet for a high cell density n~1017 bact m−3 and under the presence of a magnetic field (arrow). White scale bar is 20 μm. The droplet is identical to the one in Supplementary Movie 4. Bacteria rotate in the same direction (CW) than in the equatorial plane.

File Name: Supplementary Movie 7
Description: - **Reversal of the droplet core rotation by magnetic field reversal.** Phase-contrast images (x40 magnification, average intensity is subtracted from the movie) of a suspension of magnetotactic bacteria at high cell density, n~1017 bact m−3 at the equatorial plane of a droplet of radius R = 55 μm, under the presence of a magnetic field (direction indicated by the arrow). White scale bar is 20 μm. Magnetic field is stopped and then quickly reversed, inducing a reversal of the rotary motion of the bacteria inside the droplet.

File Name: Supplementary Movie 8
Description: **Alignment of a single bacterium with the droplet boundary.** Phase-contrast images (x40 magnification, average intensity is subtracted from the movie) of a dilute suspension of magnetotactic bacteria inside a droplet (cell density n~1014 bact m−3 . The images are separated by 1/25 s and a magnetic field of 2 mT is applied in the direction from the bottom to the top of the image. The bacterium at the boundary exhibits a wobbling motion aligning sequentially with the droplet boundary and the magnetic field direction.

File Name: Supplementary Movie 9
Description: **PIV velocity field at magnetic field reversal.** Velocity field obtained by PIV for the experiment described in Supplementary Movie 7. The black arrows indicate the magnitude and direction of the velocity vector, while the left and right panels present superimposed in colour the x and y components of the velocity, respectively. The green arrow indicates the direction of the magnetic field. At t = 17.68 s the magnetic field is turned off and at t = 19.44 s it is reversed. From t ≈ 19.7 s to t ≈ 21.4 s a transient current develops going down on the right part and up on the left part. Finally, the CCW circulation establishes from t ≈ 23.0 s onward.
